# Association of Smoking Status with Efficacy of First-line Immune Checkpoint Inhibitors in Advanced Non-small Cell Lung Cancers: A Systematic Review and Meta-analysis

**DOI:** 10.7150/jca.65374

**Published:** 2022-01-01

**Authors:** Jinchul Kim, Hyerim Ha, Jisun Park, Jinhyun Cho, Joo Han Lim, Moon Hee Lee

**Affiliations:** Department of Hematology-Oncology, Inha University College of Medicine and Hospital, Incheon, Republic of Korea.

**Keywords:** Smoking, Non-small cell lung cancer, First-line treatment, Immune checkpoint inhibitor, Meta-analysis

## Abstract

**Background:** Although smoking status has potential as a biomarker for immune checkpoint blockade in advanced non-small cell lung cancer (NSCLC), its clinical significance remains obscure. This meta-analysis aims to assess the impact of the smoking status on the efficacy of first-line immunotherapy and to find better treatment in never-smoker and ever-smoker patients.

**Methods:** We searched the MEDLINE, EMBASE, and Cochrane database for trials comparing immunotherapy with conventional chemotherapy as front-line treatment for advanced NSCLC. Random-effects models were used to pool estimates of hazard ratios (HRs) for overall survival with 95% confidence intervals (CIs). Predefined subgroup analysis was performed to investigate the difference in the efficacy between the single checkpoint blockade and checkpoint inhibitor plus chemotherapy combination in the never-smokers and current/former smokers.

**Results:** Twelve trials involving 6,446 patients were included in the analysis. A statistically significant overall survival benefit over conventional chemotherapy was found for both checkpoint inhibitor monotherapy (HR, 0.71; 95% CI, 0.59-0.85) and checkpoint inhibitor plus chemotherapy (HR, 0.75; 95% CI, 0.63-0.90) in the current/former smoker group. There was no subgroup difference between monotherapy and combination treatment (p=0.67). However, there was an inconsistent survival outcome in the never-smoker group; checkpoint blockade monotherapy did not show significantly better efficacy than chemotherapy alone (HR, 1.05; 95% CI, 0.81-1.37), but combination treatment showed an overall survival benefit (HR, 0.64; 95% CI, 0.43-0.94). A significant subgroup difference existed between monotherapy and combination therapy (p=0.04). Similarly, there was a significant difference in efficacy of monotherapy between the current/former smoker and never-smoker group (p=0.01), but the efficacy of the combination treatment was comparable between the two groups (p=0.45).

**Conclusion:** Smoking status, which is easily available information, could be used as a guide in clinical practice to choose better treatment in the front-line setting for advanced NSCLC patients.

## Introduction

Recent advancements in immune checkpoint inhibitors have transformed the treatment of advanced non-small cell lung cancer (NSCLC) using targets known immune checkpoint molecules, such as the programmed death-ligand 1 (PD-L1) and its receptor, the programmed death-1 (PD-1) 1. Through blocking the immune escape mechanism of the tumor cells, immune checkpoint inhibitors have demonstrated superior efficacy compared to conventional toxic chemotherapy. Consequently, first-line checkpoint inhibitors have been approved to replace chemotherapy in the form of monotherapy for patients with high PD-L1 expression on tumor cells or in the form of combination with chemotherapy regardless of PD-L1 expression 2-9.

However, as only a limited portion of the population with advanced NSCLC experiences long-term effects of immune checkpoint inhibition, it is still crucial to find key indicators that could maximize the efficacy of immunotherapy and guide clinical decision-making processes. PD-L1 expression is the most studied biomarker to date, and most trials demonstrated a trend between increased level of the PD-L1 expression and improved efficacy of immune checkpoint inhibitors, though its clinical usefulness still remains a topic of debate. Other researched biomarkers such as tumor mutation burden (TMB) or gene expression profiling have potential as a predictive modality, but the standardization issues and the accuracy of the prediction still need to be solved 10.

Smoking status has also been reported to have the predictive potential for immunotherapy. Previous studies that assessed the comprehensive mutational landscape of NSCLC reported that smoking exposure enhanced somatic mutations, thereby could increase tumor response to anti-PD-1/PD-L1 therapy 1, 11. Additionally, subgroup analyses of several randomized clinical trials with immune checkpoint inhibitors in first-line treatment for advanced NSCLC reported that the positive smoking history was associated with improved survival outcomes.

However, in more detail, it has been reported that there was a substantially better response to single agent checkpoint inhibitor as first-line therapy in the current/former than in the never-smoker group 2, 6, 8, but a generally similar response to checkpoint inhibitor plus chemotherapy combination treatment was reported in the two groups 3, 4. Moreover, several pooled analyses investigating the impact of smoking status on the effectiveness of immunotherapy conducted so far included various types of cancer regardless of the line of treatment 12-14, so caution is needed when interpreting these analyses due to their inherent heterogeneity. From this context, we performed a systematic review and meta-analysis to assess whether the smoking status influences the efficacy of the first-line immunotherapy treatment in patients with advanced NSCLC and affects differently between the checkpoint inhibitor monotherapy and checkpoint inhibitor plus chemotherapy combination treatment.

## Methods

This systematic review and meta-analysis was performed in accordance with the Preferred Reporting Items for Systematic Reviews and Meta-analyses (PRISMA) reporting guideline 15.

### Systematic Literature Review

Two authors (J.K. and H.H.) separately carried out a comprehensive systematic search of the literature from inception to January 15, 2021. Randomized controlled trials that compared immune checkpoint inhibitor-based treatment with chemotherapy as first-line therapy for advanced NSCLC were searched in MEDLINE, EMBASE, and the Cochrane Central Register of Controlled Trials. Searches were confined to human studies without language limitations. The main keywords used for the literature search were the *immune checkpoint inhibitors, carcinoma, non-small cell lung, and randomized controlled trial.* The details of the search strategy are described in the eMethods in the Supplement information. We also manually searched the meeting abstracts from the American Society of Clinical Oncology, European Society for Medical Oncology, and World Conference on Lung Cancer.

### Selection Criteria

We included studies meeting the following eligibility criteria: randomized controlled trial; studies including patients with advanced NSCLC (inoperable locally advanced or metastatic disease); trials comparing first-line immune checkpoint inhibitors (with or without chemotherapy) to a conventional chemotherapy agent; published as full-text articles; and studies with available data on patients' survival data according to smoking status. Studies that were retrospective or prospective observational cohort studies were excluded. Studies that compared checkpoint inhibitors with chemotherapy as second- or later-line treatment were also excluded. In addition, studies that used chemotherapy plus anti-angiogenic agent as control arms were excluded to secure maximum homogeneity.

### Data Extraction

We abstracted the most extended follow-up data including updated survival analysis in cases of multiple sources reported in the same study. The following items were extracted from each included article: trial name, treatment details, PD-L1 expression indication, study patients' clinical information (age, gender, histology), median follow-up duration, crossover rate, and the number of patients by smoking status. The hazard ratio (HR) with a corresponding 95% confidence interval (CI) for overall survival was extracted from the studies, as the primary outcome was overall survival, the time from randomization to the date of death from any cause. Two authors (J.P. and J.C.) extracted the data independently using a predefined datasheet, and the other two authors (J.H.L. and M.H.L.) resolved the inconsistencies in the extracted data. Two reviewers (J.K. and M.H.L.) evaluated the quality of the included trials using the Cochrane Collaboration risk-of-bias tool.

### Analysis Strategy

To find out whether the efficacy of checkpoint inhibitor alone and the checkpoint inhibitor plus chemotherapy combination differ according to the smoking status and to support decision-making processes in clinical practice, we planned to perform the following analyses.

1) Comparison of the efficacy of the checkpoint inhibitor monotherapy and the checkpoint inhibitor plus chemotherapy combination in the current/former smoker group and never-smoker group, respectively.

2) Comparison of the efficacy of the checkpoint inhibitor monotherapy and the checkpoint inhibitor plus chemotherapy combination, which were FDA-approved, in the current/former smoker group and never-smoker group, respectively.

3) Comparison of efficacy of the current/former smoker and never-smoker group in the checkpoint inhibitor monotherapy group and the checkpoint inhibitor plus chemotherapy combination group, respectively.

### Statistical Analysis

We conducted a meta-analysis using Review Manager, version 5.4. The inverse variance method for meta-analysis of HR was used. A random-effects models were utilized to calculate pooled HRs, corresponding 95% CIs, and P values under the assumption of clinical heterogeneity inherent in the pooled data. Study-level heterogeneity was assessed using the Q test and the I^2^ statistic. All reported *P* values were two-sided, and less than .05 were considered statistically significant.

## Results

### Search Results

**Figure 1:**
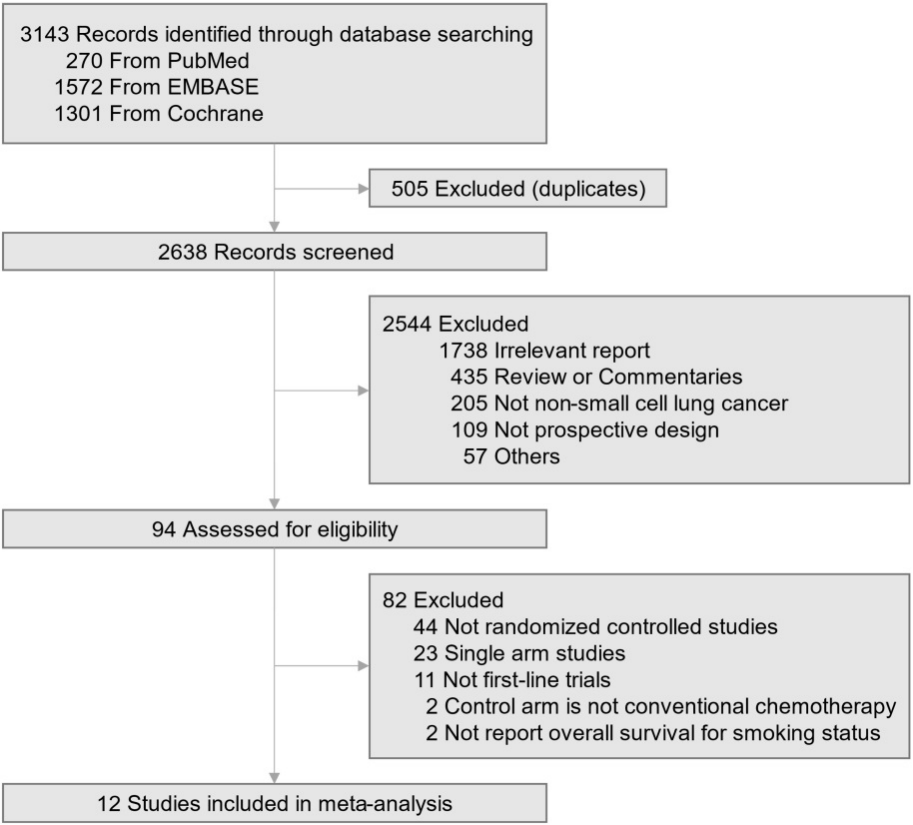
Trial selection flow diagram.

A total of 3,143 articles were searched by the initial search strategy. After the removal of 505 duplicates, 2638 studies' titles and abstracts were screened. After a full-text review of the 94 potentially eligible studies, 12 trials meeting the inclusion criteria were selected for the quantitative analysis (Fig. 1) 2-9, 16-22. All these studies enrolled a total of 6,446 patients with advanced non-small cell lung cancer, of whom 691 were never-smokers (375 in the immune checkpoint inhibitor group; 316 in the chemotherapy group). The baseline characteristics of the 12 trials are summarized in Table 1. Six trials used checkpoint inhibitor monotherapy as experimental drugs, one trial with a dual checkpoint inhibitor, and five trials with checkpoint inhibitor plus chemotherapy combination. All trials used commonly recommended chemotherapy regimens as the control group (doublet chemotherapy including cisplatin or carboplatin). PD-L1 expression eligibility varied across the trials with single agent checkpoint inhibitor. Trials that assessed the efficacy of checkpoint inhibitor and chemotherapy combination enrolled patients regardless of their PD-L1 expression level. The median age of all the included trials was in the 60s, and six studies permitted treatment crossover within the trial.

The result of the evaluation of risk of bias is provided in the Supplementary Fig. 1. As only the Keynote-189 and Keynote-407 trials were designed as double-blind and placebo-controlled, all but two studies reported a high risk of performance bias due to the open-label design. Random sequence generation was stated appropriately in most of the trials. Attrition, reporting, and other biases were not identified in any trials.

### Pooled analysis

**Figure 2:**
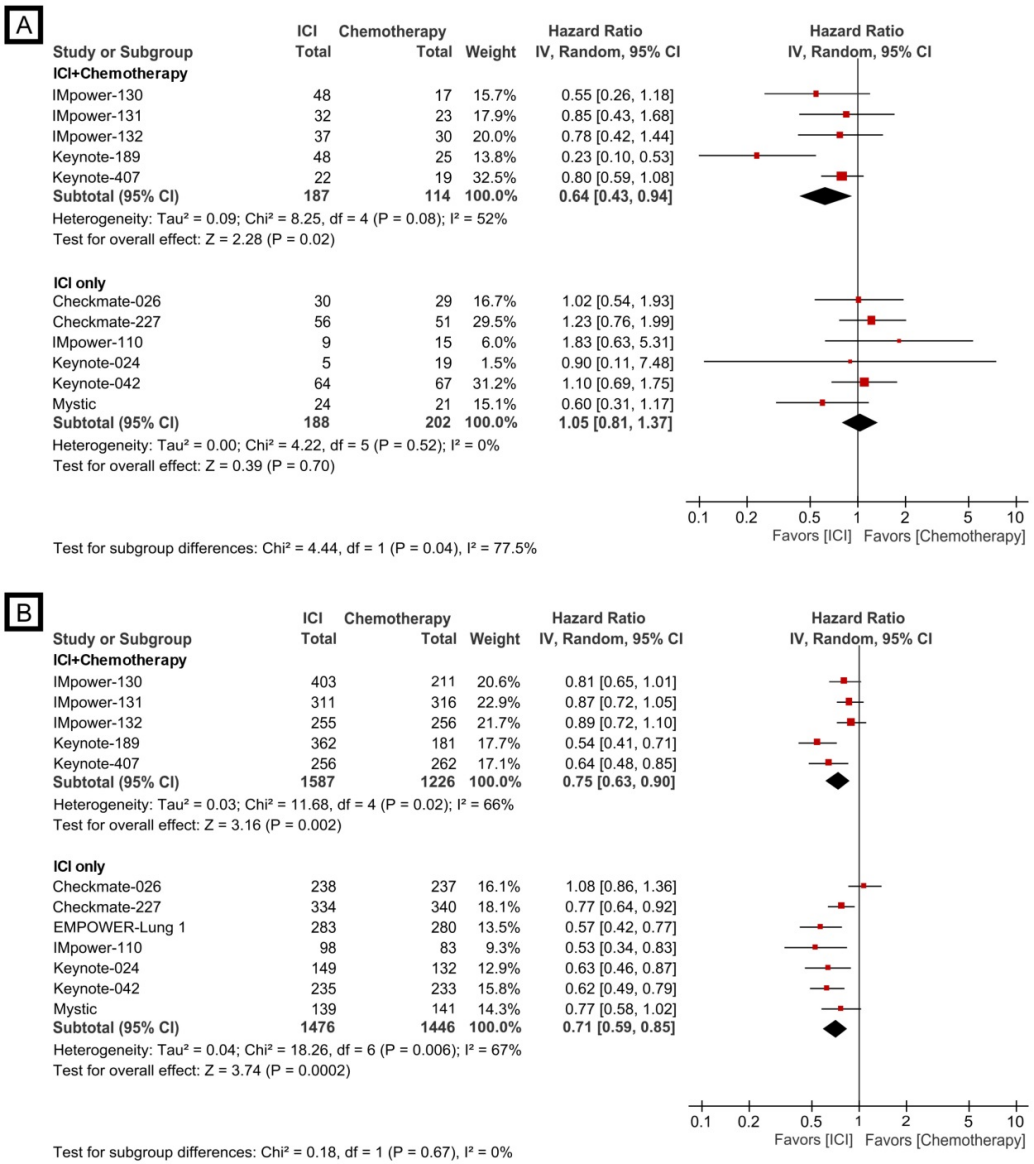
Forest plot of meta-analysis comparing checkpoint inhibitor-based treatment versus chemotherapy for overall survival by smoking status. (A) never-smoker group; (B) current/former smoker group. The size of the squares corresponds to the weight of the study in the meta-analysis. The treatment effects were calculated using a random-effects model. ICI: immune checkpoint inhibitor; CI: confidence interval.

In the never-smoker group, checkpoint inhibitor plus chemotherapy combination versus chemotherapy revealed a significantly better outcome of combination treatment (HR, 0.64; 95% CI, 0.43-0.94). However, checkpoint inhibitor monotherapy (including dual checkpoint inhibition) did not demonstrate survival benefit over chemotherapy (HR, 1.05; 95% CI, 0.81-1.37, Fig. 2A). There was a significant subgroup difference in overall survival outcome between combination and monotherapy (*p*=0.04). In the current/former smoker group, both checkpoint inhibitor plus chemotherapy combination (HR, 0.75; 95% CI, 0.63-0.90) and checkpoint inhibitor monotherapy (HR, 0.71; 95% CI, 0.59-0.85) showed a significantly better overall survival than chemotherapy (Fig. 2B). There was no difference in treatment effects between combination and monotherapy (*p*=0.67).

As several trials were conducted under different indications (e.g., PD-L1 expression level) and regimens, we performed another quantitative analysis confined to the trials with FDA-approved checkpoint inhibitor regimens. Similarly, the efficacy of checkpoint inhibitor plus chemotherapy combination and checkpoint inhibitor monotherapy versus chemotherapy were significantly different in the never-smoker group (*p*=0.03, Supplementary Fig. 1A) but were similar in the current/former smoker group (*p*=0.90, Supplementary Fig. 1B).

**Figure 3:**
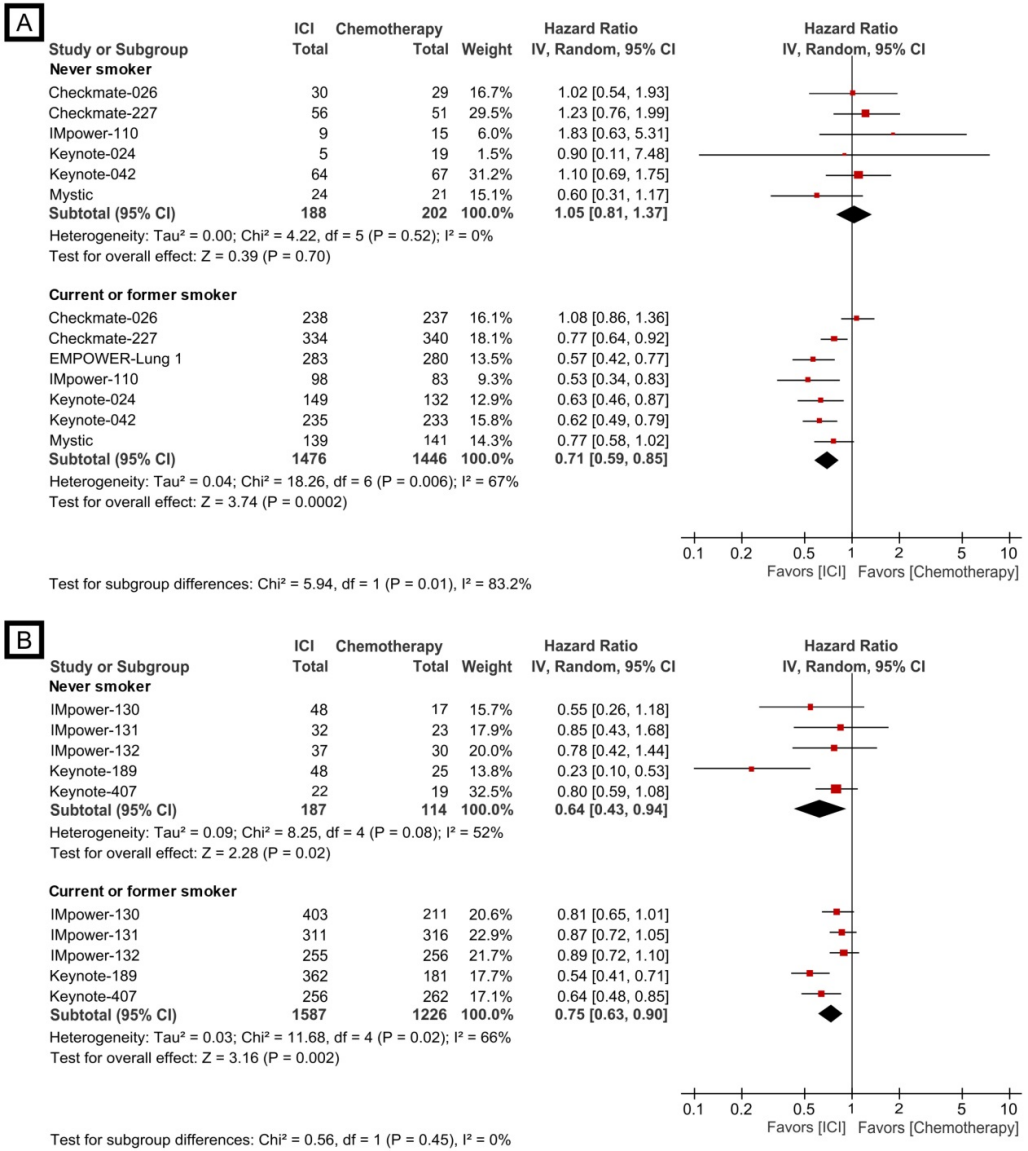
Forest plots of meta-analysis comparing checkpoint inhibitor-based treatment versus chemotherapy for overall survival according to treatment modality. (A) checkpoint inhibitor monotherapy; (B) checkpoint inhibitor plus chemotherapy combination. The size of the squares corresponds to the weight of the study in the meta-analysis. The treatment effects were calculated using a random-effects model. ICI: immune checkpoint inhibitor; CI: confidence interval.

Reversely, the efficacy of checkpoint inhibitor monotherapy and checkpoint inhibitor plus chemotherapy combination was also evaluated according to smoking status (never vs. ever-smoker). The monotherapy revealed the significantly improved treatment effect compared to cytotoxic chemotherapy in the current/former smoker group (HR, 0.71; 95% CI, 0.59-0.85), while a trend of better survival outcome for chemotherapy compared to single agent checkpoint blockade treatment in the never-smoker group was shown (HR, 1.05; 95% CI, 0.81-1.37, Fig. 3A); the subgroup analysis showed the meaningful difference between the two groups (*p*=0.01). However, the combination treatment was associated with improved overall survival compared to chemotherapy in both never (HR, 0.64; 95% CI, 0.43-0.94) and current/former smoker groups (HR, 0.75; 95% CI, 0.63-0.90, Fig. 3B), and no significant subgroup difference was observed (*p*=0.45).

**Figure 4:**
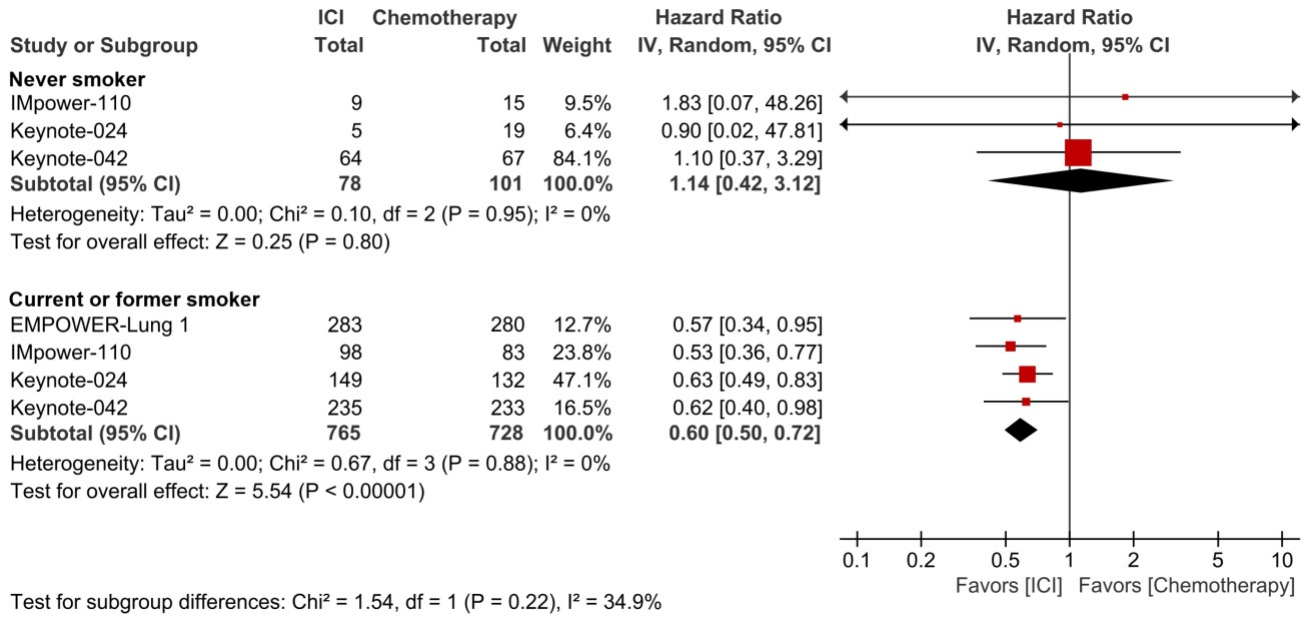
Forest plots of meta-analysis comparing checkpoint inhibitor monotherapy versus chemotherapy for overall survival by smoking status in patients with PD-L1 expression ≥50%. The size of the squares corresponds to the weight of the study in the meta-analysis. The treatment effects were calculated using a random-effects model. ICI: immune checkpoint inhibitor; CI: confidence interval.

Finally, another pooled analysis with an effort of matching the PD-L1 expression level was carried out. Checkpoint blockade monotherapy did not demonstrate the better efficacy than conventional chemotherapy in never-smoker patients with PD-L1 expression ≥50% (HR 1.14; 95% CI, 0.42-3.12), but showed a significantly superior survival outcome over chemotherapy in current/former smoker patients with PD-L1 expression ≥50% (HR 0.60; 95% CI, 0.50-0.72, Fig. 4). There was a trend of better response to checkpoint inhibitor monotherapy in the ever-smoker group than the never-smoker group, although no statistically significant subgroup difference was observed (p=0.22).

## Discussion

Despite the therapeutic efficacy of immune checkpoint inhibitors in a subset of patients, easily obtainable and consistent predictors of efficacy remain somewhat elusive. Regarding the smoking status as a predictive biomarker for immunotherapy, the results of several reports were substantially heterogeneous because of the massive scope of the previous pooled analyses, which comprised clinical trials studying multiple tumor types and using checkpoint inhibitors for different lines of treatment 12-14. Therefore, we attempted to evaluate the association of the smoking status with the effectiveness of first-line checkpoint inhibitor-based treatment in patients with advanced NSCLC. This meta-analysis demonstrated that the efficacy of the checkpoint inhibitor plus chemotherapy combination was superior to checkpoint inhibitor monotherapy in the never-smoker group but was similar in the current/former smoker group. In addition, the effect of checkpoint inhibitor monotherapy was better in the never-smoker than in the current/former smoker, but that of combination treatment was not different.

There has been a recently published study regarding the impact of smoking status on the efficacy of immunotherapy in advanced NSCLC. A meta-analysis by Dai et al**.** 23 reported that immune checkpoint inhibitors monotherapy significantly improved overall survival in ever-smoker but not in never-smoker patients compared to conventional chemotherapy, and checkpoint inhibitors plus chemotherapy combination might be the optimal selection in never-smoker. However, the high heterogeneity of the included studies was reported due to the study's inclusion criteria regardless of line of treatment. We conducted our meta-analysis including trials performed in only first-line settings, as it was reported that prior cytotoxic chemotherapy could affect cancer immunogenicity 24 and change the biological features of tumors, including PD-L1 expression level and TMB 25. In addition, to control the heterogeneity and provide more practical information in the clinical field, a pooled analysis confined to front-line treatment for advanced NSCLC was performed in this study.

Smoking can influence TMB level via the accumulation of somatic mutations by carcinogens in tobacco, leading to a higher neoantigen load 26. From this background, the idea that patients with a tumor harboring smoking signature would respond better to immunotherapy has appeared 1. On the contrary, driver mutations in NSCLC like EGFR mutation and ALK, ROS1, RET, and NTRK rearrangements are more prevalent in non-smokers, and most of these molecular alterations are associated with low TMB level, which may partly explain the lower efficacy of immune checkpoint inhibitors in never-smoker patients with advanced NSCLC 27, 28.

Although only a small number of patients without a smoking history were included in each trial, it has been reported that there was a noticeable different response to checkpoint inhibitor monotherapy as front-line treatment between the current/former and never-smoker 2, 6, 8. Based on this observation, the EMPOWER-Lung 1 trial, which assessed the efficacy of the PD-1 inhibitor cemiplimab, even set a never-smoking history as one of the ineligibility criteria for study participation 9. Moreover, in a study that assessed the clinical activity of single PD-1 blockade in never, light, and heavy smoker patients with PD-L1 expression ≥50%, heavy smokers demonstrated better response and longer progression-free survival compared to never/light smokers 29. Another study also confirmed that ever-smokers with PD-L1 expression ≥50% receiving first-line checkpoint inhibitor monotherapy experienced improved overall survival compared to never-smokers 30. Finally, Wang et al. 31 provided evidence that improved clinical outcome to checkpoint inhibitor monotherapy was statistically significant with increased tobacco exposure, which could be a more detailed prognostic information than categorized smoking history (never vs. current/former).

However, in contrast, trials of the checkpoint inhibitor and chemotherapy combination mostly did not demonstrate a significant difference in treatment effectiveness between the two groups; subgroup analysis according to TMB level also showed a similar survival benefit of combination treatment in the TMB-high and TMB-low subgroups 32. It seems that the combination therapy of immunotherapy and cytotoxic chemotherapy may offset the little effect of checkpoint inhibitor monotherapy in TMB-low tumors in non-smoker patients, but further studies are warranted as the exact mechanism has not been yet well studied. Conclusively, it would be suggested that although smoking status could serve as a predictive biomarker for checkpoint inhibitor monotherapy, it is not suitable for combination therapy.

Our study's major limitation was the discrepancy of PD-L1 expression indication among the included trials. Most of the trials with checkpoint inhibitor monotherapy enrolled patients harboring PD-L1 expression more than 50%, and trials with combination treatment included patients regardless of PD-L1 expression. What we found in this study was that checkpoint inhibitor monotherapy was significantly less effective than the combination treatment in the never-smoker subgroup; considering the efficacy analysis according to the PD-L1 expression level, it is unlikely that the never-smoker subpopulation with PD-L1 expression ≥50% is less responsive to the checkpoint inhibitor plus chemotherapy combination than the subpopulation with low PD-L1 expression. Additionally, subgroup analysis of the Keynote-042 trial reported that the difference in efficacy of pembrolizumab between the patients with the PD-L1 expression ≥1% and ≥50% in the never-smoker group was almost similar 6. Taking these results together, the fact that the indication of PD-L1 expression was different for each trial is not considered to disturb the reliability of the conclusion of this study.

Additionally, we encountered a few other limitations during this study. First, due to the intrinsic limitation for analysis of subgroup population, there could be slightly unbalanced patient distribution and features between the intervention and control according to smoking status. Second, we could not perform subgroup analysis to assess heterogeneity within combination therapy and monotherapy group due to the scarcity of included studies. However, the current analysis is a study that performed a predefined subgroup analysis by the smoking status, and additional analysis confined to FDA-approved trials was carried out to reflect the actual situation and control various sources of heterogeneity. Third, the current and former smokers were grouped together and analyzed as a single group, and detailed analysis according to the quantitative information on smoking was not possible. Despite these limitations, the present analysis provided evidence that the smoking status, which is easily obtainable information from patients, could be used as a guide to help with clinical decision-making processes in front-line treatment for advanced NSCLC.

In summary, the current study suggests that checkpoint inhibitor monotherapy should be cautiously used in first-line systemic treatment for never-smoker patients with advanced NSCLC, and checkpoint inhibitor and chemotherapy combination treatment should be considered first in this group.

## Supplementary Material

Supplementary methods and figures.

## Figures and Tables

**Table 1:** Characteristics of the Included Randomized Controlled Trials.

						No. of Patients					
						ICI Group		Chemotherapy Group			
Trial	Intervention vs Control	PD-L1 expression	Age, Median (Range), year	FemaleNo. (%)	Squamous HistologyNo. (%)	Never Smoker	Total	Never Smoker	Total	Follow-up Duration, Median, mo.	Crossover rate (%)
IMpower-110^8^	Atezolizumab vs Chemotherapy	TC3 or IC3	63 (33-87)	62 (30.2)	50 (24.4)	9	107	15	98	15.7	Not permitted
Keynote-024^2^,^17^	Pembrolizumab vs Chemotherapy	≥50%	66 (33-90)	118 (38.7)	56 (18.4)	5	154	19	151	25.2	43.7
Keynote-042^6^	Pembrolizumab vs Chemotherapy	≥50%	64 (57-69, IQR)	184 (30.7)	221 (36.9)	64	299	67	300	12.8	Not permitted
Checkmate-026^16^	Nivolumab vs Chemotherapy	≥1%	64 (29-89)	209 (39.1)	130 (24.1)	30	271	29	270	13.5	60
Mystic^21^	Durvalumab vs Chemotherapy	TC≥25%	64 (32-85)	106 (32.6)	104 (32.0)	24	163	21	162	30.2	Not permitted
EMPOWER-Lung 1^9^	Cemiplimab vs Chemotherapy	≥50%	64 (57-70, IQR)	84 (14.9)	243 (43.2)	0	283	0	280	10.9	74
Checkmate-227^5^	Nivolumab+Ipilimumab vs Chemotherapy	≥1%	64 (26-87)	278 (35.1)	233 (29.4)	56	396	51	397	29.3 (minimum)	Not permitted
Keynote-189^3^,^18^	Pembrolizumab+Chemotherapy vs Chemotherapy	All	65 (34-84)	253 (41.1)	0 (0)	48	410	25	206	23.1	32.5 (pembrolizumab monotherapy)
Keynote-407^4^,^20^	Pembrolizumab+Chemotherapy vs Chemotherapy	All	65 (29-88)	104 (18.6)	559 (100)	22	278	19	281	14.3	31.7 (pembrolizumab monotherapy)
IMpower-130^7^	Atezolizumab+Chemotherapy vs Chemotherapy	All	64 (18-86)	279 (41.1)	0 (0)	48	451	17	228	19.2	41 (atezolizumab monotherapy)
IMpower-131^19^	Atezolizumab+Chemotherapy vs Chemotherapy	All	65 (23-86)	126 (18.4)	683 (100)	32	343	23	340	26.8	Not permitted
IMpower-132^22^	Atezolizumab+Chemotherapy vs Chemotherapy	All	64 (31-85)	194 (33.6)	0 (0)	37	292	30	286	14.8	Not permitted

ICI: immune checkpoint inhibitor; PD-L1: programmed death-ligand 1; TC: tumor cells; IC: immune cells; IQR: interquartile range.
