# Supplementary material for: Association of Smoking Status with Efficacy of First-line Immune Checkpoint Inhibitors in Advanced Non-small Cell Lung Cancers: A Systematic Review and Meta-analysis
Source: J Cancer. 2022 Jan 1;13(2):364–72. doi: 10.7150/jca.65374 (PMC8771515; doi:10.7150/jca.65374)

Supplementary Online Content

Association of Smoking Status with Efficacy of First-line Immune Checkpoint Inhibitors in Advanced Non-small Cell Lung Cancers: A Systematic Review and Meta-analysis

Jinchul Kim*, Hyerim Ha*, Jisun Park, Jinhyun Cho, Joo Han Lim, and Moon Hee Lee

eMethods. Search strategies for PubMed, EMBASE, Cochrane database

eFig.1. Risk of bias of included studies

eFig.2. Forest plots of meta-analysis comparing checkpoint inhibitor-based treatment versus chemotherapy for overall survival by smoking status, which confined to trials with FDA-approved regimens.

eMethods. Search strategies for PubMed, EMBASE, Cochrane database

PubMed search details: 270 results

(("carcinoma, non-small-cell lung"[Mesh] OR "NSCLC"[Title/Abstract] OR "Non-Small Cell Lung Cancer"[All Fields]) AND ("checkpoint inhibitor"[Title/Abstract] OR "pembrolizumab"[Title/Abstract] OR "nivolumab"[Title/Abstract] OR "atezolizumab"[Title/Abstract] OR "durvalumab"[Title/Abstract] OR "ipilimumab"[Title/Abstract] OR "cemiplimab"[Title/Abstract])) AND (("random allocation"[MeSH Terms] OR "randomized"[All Fields] OR "randomised"[All Fields] OR "randomly"[All Fields]) AND ("clinical trials as topic"[MeSH Terms] OR "trial"[Title/Abstract] OR "study"[Title/Abstract]))

EMBASE: 1,572 results

('non-small cell lung cancer'/exp OR 'nsclc':ab,ti OR ('lung':ab,ti AND ('non small cell':ab,ti OR 'non-small-cell':ab,ti OR 'non-small cell':ab,ti))) AND ('checkpoint inhibitor':ab,ti OR 'pembrolizumab':ab,ti OR 'nivolumab':ab,ti OR 'atezolizumab':ab,ti OR 'durvalumab':ab,ti OR 'ipilimumab':ab,ti OR 'cemiplimab':ab,ti) AND 'human'/de AND ('clinical trial'/de OR 'randomized controlled trial'/exp)

Cochrane database: 1,301 results

#1 "carcinoma, non- small-cell lung" OR "non-small cell lung cancer" OR "nsclc"

#2 "checkpoint inhibitor" OR "pembrolizumab" OR "nivolumab" OR "atezolizumab" OR "durvalumab" OR "ipilimumab" OR "cemiplimab"

#3 trials

#1 AND #2 AND #3

eFigure 1. Risk of bias of included studies

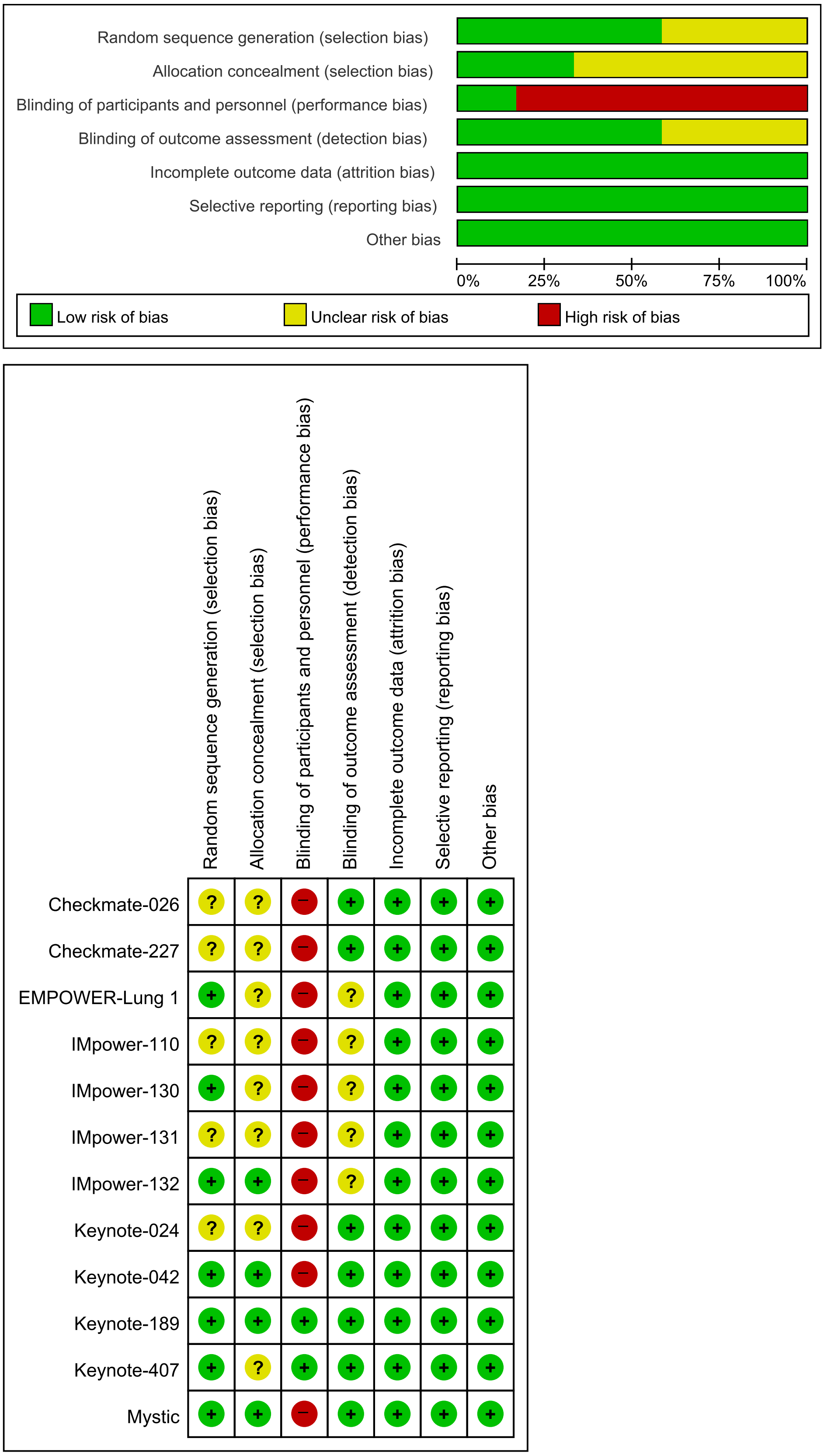

eFigure 2. Forest plots of meta-analysis comparing checkpoint inhibitor-based treatment versus chemotherapy for overall survival by smoking status, which confined to trials with FDA-approved regimens. (A) never-smoker group; (B) current/former smoker group. The size of the squares corresponds to the weight of the study in the meta-analysis. The treatment effects were calculated using a random-effects model.

A

Study or Subgroup	ICI Total	Chemotherapy Total	Weight	Hazard Ratio IV, Random, 95% CI
**ICI+Chemotherapy**				
IMpower-130	48	17	30.0%	0.55 [0.26, 1.18]
Keynote-189	48	25	27.8%	0.23 [0.10, 0.53]
Keynote-407	22	19	42.2%	0.80 [0.59, 1.08]
**Subtotal (95% CI)**	**118**	**61**	**100.0%**	**0.51 [0.25, 1.03]**
Heterogeneity: Tau² = 0.29; Chi² = 7.76, df = 2 (P = 0.02); I² = 74%				
Test for overall effect: Z = 1.88 (P = 0.06)				
**ICI only**				
Checkmate-227	56	51	43.3%	1.23 [0.76, 1.99]
IMpower-110	9	15	8.7%	1.83 [0.63, 5.31]
Keynote-024	5	19	2.2%	0.90 [0.11, 7.48]
Keynote-042	64	67	45.8%	1.10 [0.69, 1.75]
**Subtotal (95% CI)**	**134**	**152**	**100.0%**	**1.20 [0.88, 1.65]**
Heterogeneity: Tau² = 0.00; Chi² = 0.82, df = 3 (P = 0.85); I² = 0%				
Test for overall effect: Z = 1.14 (P = 0.25)				

Test for subgroup differences: Chi² = 4.77, df = 1 (P = 0.03), I² = 79.0%

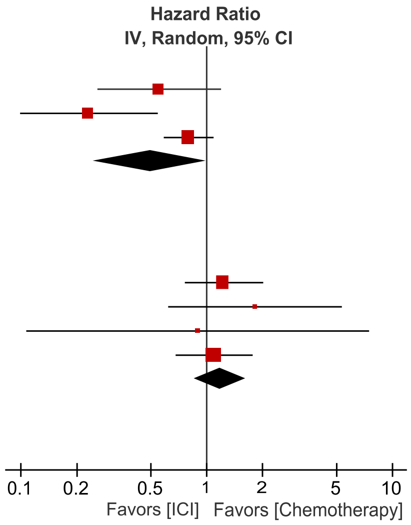

B

Study or Subgroup	ICI Total	Chemotherapy Total	Weight	Hazard Ratio IV, Random, 95% CI
**ICI+Chemotherapy**				
IMpower-130	403	211	37.0%	0.81 [0.65, 1.01]
Keynote-189	362	181	32.0%	0.54 [0.41, 0.71]
Keynote-407	256	262	31.0%	0.64 [0.48, 0.85]
**Subtotal (95% CI)**	**1021**	**654**	**100.0%**	**0.66 [0.52, 0.84]**
Heterogeneity: Tau² = 0.03; Chi² = 5.19, df = 2 (P = 0.07); I² = 61%				
Test for overall effect: Z = 3.34 (P = 0.0008)				
**ICI only**				
Checkmate-227	334	340	43.3%	0.77 [0.64, 0.92]
IMpower-110	98	83	10.0%	0.53 [0.34, 0.83]
Keynote-024	149	132	18.0%	0.63 [0.46, 0.87]
Keynote-042	235	233	28.7%	0.62 [0.49, 0.79]
**Subtotal (95% CI)**	**816**	**788**	**100.0%**	**0.67 [0.58, 0.78]**
Heterogeneity: Tau² = 0.00; Chi² = 3.70, df = 3 (P = 0.30); I² = 19%				
Test for overall effect: Z = 5.25 (P < 0.00001)				

Test for subgroup differences: Chi² = 0.02, df = 1 (P = 0.90), I² = 0%

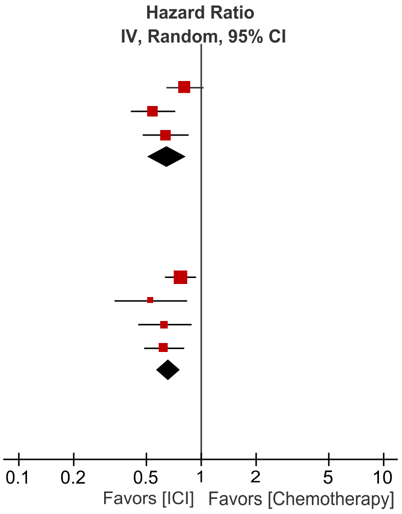

Supplement: Supplementary file 1 — Supplementary methods and figures. [file jcav13p0364s1.pdf]
